# Comparison of outcomes following prepectoral and subpectoral implants for breast reconstruction in patients with breast cancer

**DOI:** 10.3389/fonc.2024.1499710

**Published:** 2025-01-07

**Authors:** Jun Zhang, Ran An, Zhi-Hao Yu, Li Zhang

**Affiliations:** ^1^ Thyroid and Breast Medical Center, Weifang People’s Hospital, Shandong Second Medical University, Weifang, Shandong, China; ^2^ Department of Breast Disease, Weifang Maternal and Child Health Hospital, Weifang, Shandong, China; ^3^ The First Department of Breast Cancer, Tianjin Medical University Cancer Institute and Hospital, National Clinical Research Center for Cancer, Tianjin, China

**Keywords:** breast reconstruction, prepectoral implants, subpectoral implants, breast cancer, comparison of outcomes

## Abstract

**Background:**

In recent years, different approaches to implant-based breast reconstruction have increasingly become an important option to meet both the treatment and postoperative aesthetic needs of breast cancer patients. This study selected two commonly used techniques for the prepectoral approach: single-incision, gas-inflated endoscopic prepectoral breast reconstruction (SIE-BR) and open prepectoral implant-based breast reconstruction (C-BR), as well as a commonly used technique for the subpectoral approach: open subpectoral implant-based breast reconstruction (SI-BR). By comparing the clinical efficacy and aesthetic outcomes of these three techniques in the treatment of breast cancer patients, this study aims to summarize the advantages of the prepectoral approach.

**Methods:**

This study screened the clinicopathological data of a total of 136 breast cancer patients from January 2023 to December 2023. Among them, 38 patients underwent SIE-BR, 51 patients underwent C-BR, and 47 patients underwent SI-BR. The patient characteristics, intraoperative and postoperative conditions were analyzed in detail, and satisfaction was assessed using the BREAST-Q questionnaire.

**Results:**

The SIE-BR group had the longest surgery time, followed by the SI-BR group, with the C-BR group having the shortest surgery time. The C-BR group had the least blood loss, while the SIE-BR group had the most. The C-BR group also had the lowest drainage volume, and the SIE-BR group had the highest. Patients were categorized into a prepectoral implant-based reconstruction group (PIBR) and a subpectoral implant-based reconstruction group (SIBR). None of the patients experienced implant loss or flap necrosis. The PIBR group had significantly lower rates of wound infection, capsular contracture, and chest muscle pain compared to the SIBR group. The rates of wound dehiscence and implant wrinkling were statistically similar between the two groups. BREAST-Q scores indicated similar satisfaction in terms of breast appearance and sexual life between the groups, but the PIBR group showed significantly better scores in physical health (chest muscle function preservation) and mental health. Additional advantages of the prepectoral approach, including less postoperative pain, reduced movement-related deformity, and shorter surgery time, have contributed to the steady growth of this technique in recent years.

**Conclusion:**

The three implant-based breast reconstruction techniques mentioned above are safe and feasible. Compared to the previously more common subpectoral approach, the prepectoral approach improves patients’ postoperative physical and psychological comfort, making it an ideal surgical option.

## 1 Introduction

Breast cancer is a serious issue affecting the physical and mental health of women in modern society, with the highest incidence rate and being the leading cause of cancer-related deaths (1). As a crucial part of systemic treatment for malignant breast tumors, traditional surgical approaches often result in significant physical and psychological trauma for patients. With the improvement of living standards, patients are placing increasing emphasis on the aesthetic outcomes after surgery. Consequently, subcutaneous mastectomy combined with immediate implant-based breast reconstruction via different approaches has become a common choice. Compared to autologous tissue breast reconstruction, implant-based breast reconstruction offers the advantages of simpler surgical procedures, less trauma, shorter surgery time, quicker postoperative recovery, and fewer complications. More importantly, it avoids the functional impact caused by damage to donor site tissues in autologous breast reconstruction (2, 3). The implant pocket can be placed either in front of the pectoralis major muscle (prepectoral) or behind it (subpectoral). However, since its introduction in the 1970s, prepectoral implant-based breast reconstruction gradually fell out of favor due to higher rates of infection, capsular contracture, wound dehiscence, and poor cosmetic outcomes, and was largely replaced by the subpectoral approach (4). In recent years, however, advances in surgical techniques and related materials have helped prepectoral reconstruction overcome these issues, leading to its increased use (5–7). With the widespread adoption of minimally invasive endoscopic surgery in modern surgery (8–10), we also introduced single-incision, gas-inflated endoscopic prepectoral implant-based breast reconstruction. Its advantages include a concealed incision and scar-free breast skin surface, thereby reducing the risk of wound dehiscence and implant exposure (11–14). Of course, endoscopic-assisted techniques also have notable drawbacks, such as longer operative times and the need for additional equipment (13–15). This study, a retrospective cohort study involving 136 patients, aims to compare the clinical efficacy of these three surgical techniques.

## 2 Materials and methods

### 2.1 Patient selection

Due to the limited adoption of pre-pectoral breast reconstruction in previous years, the procedure has become more prevalent with advancements in understanding of the disease, anatomical knowledge, and recent research reports. Consequently, starting from last year, pre-pectoral breast reconstruction has been increasingly performed. This study retrospectively analyzed breast cancer patients who visited the Department of Breast Oncology at Tianjin Cancer Hospital from January 2023 to December 2023. Inclusion criteria: Patients with a desire for breast reconstruction who underwent either pre-pectoral or sub-pectoral implant breast reconstruction. Exclusion criteria: Patients with infection at the site of the breast surgery incision; Patients with severe psychological or psychiatric issues; Patients with Paget’s disease, locally advanced breast cancer, and/or distant metastasis; Patients who cannot undergo breast reconstruction or bear the potential risks of complications; Patients with imaging evidence of suspected pectoral and/or skin involvement; Patients with serious diseases who cannot tolerate anesthesia or surgery. A total of 136 patients were selected (**Table 1**). Based on the postoperative pathology results, subsequent treatment plans, including chemotherapy, radiotherapy, targeted therapy, and endocrine therapy, were formulated. Prior to surgery, all relevant examinations were completed, and all patients signed an informed consent form. Satisfaction was assessed postoperatively using the BREAST-Q questionnaire. Follow-up continued through outpatient visits and phone calls until June 2024.

**Table 1:** Clinical data of the study patients.

Variable	PIBR		SIBR (N= 47)	P Variable
	C-BR (N= 51)	SIE-BR (N= 38)		
Age (years)				0.670
Mean Range	45.31±9.58	43.05±5.02	44.26±9.53	
Neoadjubant chemotherapy, n (%)	21.6	23.7	21.3	0.960
Tumor location, n (%)				0.241
Upper Outer Quadrant	52.9	47.4	59.6	
Upper Inner Quadrant	15.7	23.7	6.4	
Lower Outer Quadrant	17.6	15.8	19.1	
Lower Inner Quadrant	11.8	5.3	4.3	
Borderline	2	7.9	1.06	
SLNB or ALND, n (%) SLNB only	88.2	78.9	70.2	0.088
ALND after SLND, n (%)	11.8	21.1	29.8	
pT staging, n (%)				0.250
pT1	33.33	50.00	31.91	
pT2	62.75	47.37	57.45	
pT3	3.92	2.63	10.64	
Radiation after surgery, n (%)	11.8	21.1	29.8	0.088
Degree of tumor differentiation, n (%)				0.476
G0	17.65	18.42	25.53	
G1	5.88	7.9	6.38	
G2	56.86	52.63	61.70	
G3	19.61	21.05	6.38	
ER, n (%)				0.654
Positive	88.235	81.579	82.979	
Negative	11.765	18.421	17.021	
PR, n (%)				0.753
Positive	78.431	84.211	82.979	
Negative	21.569	15.789	17.021	
HER-2, n (%)				0.102
Positive	19.61	28.95	10.64	
Negative	80.39	71.05	89.36	
Ki67	25(15,40)	20(14.25,41.25)	20(10,30)	0.587

ER estrogen receptor; PR progesterone receptor; HER2 human epidermal growth factor receptor type 2; SLNB sentinel lymph node biopsy; ALND axillary lymph node; dissection; pT staging pathological tumor staging; pN staging pathological lymph node staging;SIBR subpectoral Implants for Breast Reconstruction; PIBR prepectoral Implants for Breast Reconstruction; SIE-BR single-port insufflation endoscopic for breast reconstruction; C-BR conventional breast reconstruction.

### 2.2 Surgical approach selection

The choice of surgical method should comprehensively consider the patient’s pathological type and anatomical characteristics. The main selection criteria are as follows:

#### 2.2.1 SI-BR

①Early-stage breast cancer patients who are unsuitable or unwilling to undergo breast-conserving surgery, with a clinical tumor size (assessed by physical examination or imaging) of <5 cm, and clinically negative axillary lymph nodes or clinically positive nodes with negative biopsy results.

②Tumors that are superficial, close to the skin, or involve the nipple-areola complex.

③Pathological type of invasive micropapillary carcinoma (this type tends to exhibit lymphovascular tumor emboli invading subcutaneous fat).

④Skin damage or excessively thin subcutaneous fat.

⑤Patients in relatively poor health, such as those with poorly controlled diabetes or who smoke.

#### 2.2.2 C-BR

①Early-stage breast cancer patients who are unsuitable or unwilling to undergo breast-conserving surgery, with a clinical tumor size (assessed by physical examination or imaging) of <5 cm, and clinically negative axillary lymph nodes or clinically positive nodes with negative biopsy results.

②Good skin condition and adequate subcutaneous fat thickness.

③Tumors located relatively far from subcutaneous fat, with imaging showing no obvious spiculations toward the skin or subcutaneous fat.

#### 2.2.3 SIE-BR

Suitable for patients meeting the criteria for C-BR who also desire no surgical scars on the breast surface.

### 2.3 Surgical procedures

#### 2.3.1 Preoperative marking

The patient assumes a standing position, and the following landmarks are marked: the breast contour, the breast-infra-mammary fold, the surface projection of the lesion, and the estimated location of the sentinel lymph node (approximately at the junction of the posterior edge of the pectoralis major and the upper outer edge of the breast, close to the back).

#### 2.3.2 Surgical positioning and preparation

The patient is positioned supine, with the affected upper limb abducted to 90 degrees and the shoulder elevated on a support. The disinfected area should cover the affected limb so that it can be raised to the forehead during the procedure, facilitating the dissection of the level III lymph nodes and providing better exposure of the axilla during endoscopic surgery.

#### 2.3.3 Implant selection

Based on the patient’s breast size and personal preferences, specific implant sizes are selected using a combination of weight measurement and diameter measurement methods. Diameter Measurement Method: Base Diameter Measurement: Measure the distance from the midline at the anterior chest to the axillary anterior line (X) using calipers. Use a V-shaped caliper to measure the thickness of the lateral breast tissue (Y) and medial breast tissue (Z). The implant base diameter is calculated as X - (Y/2 + Z/2). Implant Height (Low, Medium, High, or Extra High): Measure the distance from the sternal notch to the nipple (SN) and the distance between the nipples (NN). If (SN - NN) is 0–2 cm, choose a medium-high implant. If (SN - NN) < 0, indicating breast expansion or the nipple is positioned outward and upward, choose a low-high implant. If (SN - NN) > 2 cm, indicating the breast is positioned inward and downward, choose a full-height or extra-high implant. Implant Projection: Choose a full-projection implant if there is significant sagging, skin looseness, or if a larger implant volume is desired; otherwise, choose a medium or low-projection implant. Weight Measurement Method: During the procedure, after removing the breast tissue, place it on a high-precision electronic scale to obtain its weight. This weight roughly corresponds to the volume of the implant. The final implant size is determined by combining the results of both methods (Mentor MemoryGel Xtra Breast Implants; Mentor Worldwide LLC, USA), with the majority being textured anatomical shapes and a few being textured round shapes.

#### 2.3.4 Sentinel lymph node biopsy

For patients with clinically negative axillary lymph nodes, sentinel lymph node biopsy (SLNB) is recommended as the primary approach. If SLNB or preoperative biopsy results are positive, axillary lymph node dissection (ALND) is recommended. For open surgery, a short incision of approximately 3 cm can be made at the location of the suspected sentinel lymph node, with the incision extended if ALND is needed. For laparoscopic surgery, a single-port incision of approximately 6 cm can be made at the axillary fold (with the anterior edge not extending beyond the anterior axillary line to ensure it is covered when the patient’s arm is in a hanging position). This incision allows for direct visualization and completion of SLNB or ALND, as well as laparoscopic subcutaneous gland removal and anterior chest muscle prosthesis implantation.

Fifteen minutes before surgery, inject blue dye intradermally along the edge of the areola. After obtaining the sentinel lymph node through the axillary incision, decide whether to perform axillary lymph node dissection based on the frozen section pathology results.

#### 2.3.5 Surgical technique

##### 2.3.5.1 SIE-BR

First, make an incision in the axilla and, under direct vision, attempt to dissect as much of the space behind the pectoralis major muscle as possible. Insert a disposable incision retractor/protector (80/90) and connect it to a disposable single-port surgical system. Inflate with CO2 at a pressure of 8-10 mmHg to create the operative space. Continue to dissect the space behind the pectoralis major muscle with an electrocautery hook, exposing the surrounding ligaments of the breast from the subclavian ligament up, the sternal ligament medially, and the triangular fascial bundle downward. During this process, be cautious as the glandular tissue edge may extend beyond the preoperative markings on the skin. Ensure no residual glandular tissue remains, and protect the sternal ligament as it is crucial for the shape of the reconstructed breast fold. Additionally, do not extend the dissection beyond the triangular fascial bundle and horizontal ligament to preserve the breast fold. Next, dissect the subcutaneous plane of the breast. Using the disposable single-port surgical system, inject adrenaline saline (205 ml of 0.9% sodium chloride solution + 1 ml of 0.1% adrenaline) between the skin and glandular tissue to establish a preliminary space and reduce bleeding during subsequent steps. After injection, use a long-handled scalpel to sharply dissect along the space between the skin and glandular tissue, preserving the thickness of the skin flap while ensuring tumor safety. Reinsert the disposable single-port surgical system and disposable incision retractor/protector (80/90). After disconnecting the surrounding ligaments with the electrocautery hook, remove the entire glandular tissue. Mark the orientation of the excised specimen and send the tissue from the area behind the nipple for frozen section pathology. Weigh the excised glandular tissue and use the preoperative measurements obtained through the diametrical method to select an appropriate implant. Place the implant into the previously dissected space in front of the pectoralis major muscle and adjust its position. Because there is no incision on the breast surface, the skin’s mechanical integrity is generally well-preserved, so additional artificial patches are usually not needed unless the skin flap is very thin or the implant size is excessively large. Before implanting the prosthesis, ensure thorough hemostasis, change gloves for both the surgeon and assistant, soak the implant in antibiotics, and prevent contact between the implant and sharp instruments or dressings. Wash the surgical area with an iodine-containing solution and place two drains at the lower fold near the anterior axillary line. Position one drain on the inner side of the implant and the other on the outer side. Close the incision layer by layer.

##### 2.3.5.2 C-BR

First, perform the sentinel lymph node biopsy or axillary lymph node dissection through the axillary incision. This procedure is similar to the steps for open chest muscle posterior implant breast reconstruction, but it is important to preserve the suspensory ligament of the breast, especially avoiding excessive dissection towards the sternum to protect the sternal ligament, which helps maintain the contour of the breast fold. Dissect the triangular fascial bundle downward towards the head side, protecting the distal part of the triangular fascial bundle and the horizontal ligament, and preserve the lower breast fold. Assess the size of the implant. If the implant volume is large, or if there are risk factors such as excessive skin tension or thin flaps after suturing the incision, use additional artificial patch materials (TiLop Bra breast soft tissue reinforcement patch; PFM Medical Group, Germany) to construct the implant pocket. Place one drain above and one below the implant, with an additional puncture site near the lower fold close to the anterior axillary line for high negative pressure drainage. Close the incision with intradermal sutures.

##### 2.3.5.3 SI-BR

First, perform the sentinel lymph node biopsy or axillary lymph node dissection through the axillary incision. Make a radial incision on the lateral side of the affected breast, incise the skin, and inject adrenaline saline (250 ml of 0.9% sodium chloride solution + 1 ml of 0.1% adrenaline) into the subcutaneous tissue. Use a scalpel to separate the skin flap, ensuring that the flap near the tumor is kept thin. Dissect the incision from the inner side to the sternal border, outer side to the anterior border of the latissimus dorsi, upward to the lower edge of the clavicle, and downward to the upper edge of the rectus abdominis sheath. Make a vertical incision through the subcutaneous tissue to reach the surface of the pectoralis major muscle. Completely excise the glandular tissue from the inner upper to the outer lower side, taking care to preserve the pectoralis major fascia. Send the specimen for routine pathology. Remove the tissue behind the nipple, mark its orientation, and send it for frozen section pathology. Remove the large ducts inside the nipple and send them for routine pathology. At 3 cm from the midline of the clavicle along the direction of the muscle fibers, make an incision in the fascia on the lateral border of the pectoralis major muscle. Dissect the fascia from the inside out to the anterior axillary line, creating a fascial flap. Lift the pectoralis major muscle and dissect the space between the pectoralis major and minor muscles, extending inward to the sternal border and downward to the breast fold. For smaller implants, place them directly into this cavity and cover the lateral surface of the implant with the adjacent fascial flap. Use intermittent sutures to attach the lateral edge of the pectoralis major muscle to the adjacent fascial flap. For larger implants, retain the superficial pectoralis major fascia and the adjacent serratus anterior fascia during surgery. Dissect the pectoralis major muscle from its attachment to the rib cage, and suture the muscle end to the fascia to cover the implant, which enhances the aesthetic result of the reconstructed breast and eliminates the need for an artificial patch. Place one drain above and one below the pectoralis major muscle, and one more on the surface of the muscle. All drains should be placed near the lower fold close to the anterior axillary line for high negative pressure drainage. Close the incision with intradermal sutures.

### 2.4 Follow-up and data analysis

2.4.1 Collect basic information on the three groups of patients, including age, body mass index, size of the lesion on ultrasound, and postoperative routine pathology.
2.4.2 Observe and compare the three groups regarding surgical duration, intraoperative blood loss, drainage volume in the first three postoperative days, and total hospitalization costs.
2.4.3 Use the BREAST-Q outcome scale to assess breast satisfaction, psychosocial status, chest wall condition, and sexual health.
2.4.4 Conduct survival data follow-up through medical records, outpatient visits, phone calls, WeChat, and questionnaires, recording occurrences of local recurrence or distant metastasis, as well as severe postoperative complications such as wound dehiscence, implant removal, and infection. Follow-up concluded on June 30, 2024.

### 2.5 Statistical methods

Numerical variables were described using mean ± standard deviation (for normal distribution) or median (first quartile, third quartile) (for non-normal distribution). Differences in patient age, surgical time, hospitalization costs, BREAST-Q scores, blood loss, total drainage volume, and routine pathological Ki67 index were compared using independent samples t-test (for normal distribution) or Mann-Whitney U test (for non-normal distribution). Categorical variables, such as lesion location, tumor stage, hormone receptor status, tumor histological grade, Her-2 status, use of neoadjuvant chemotherapy, and axillary dissection, were presented as absolute numbers and percentages. Chi-square tests were used to compare baseline data differences between the two groups. All statistical analyses were performed using SPSS version 26.0 (IBM Corp., Armonk, NY, USA). A significance threshold was set at p < 0.05.

## 3 Results

### 3.1 Patient demographic characteristics

The open prepectoral implant group (C-BR) comprised 51 patients, the single-port inflatable endoscopic breast reconstruction group (SIE-BR) included 38 patients, and the open subpectoral implant group (SI-BR) had 47 patients. There were no statistical differences among the three groups in terms of age, tumor stage, routine pathological grading, tumor immunohistochemical status (ER, PR, Her-2, Ki67 index), tumor location, total hospitalization costs, or the proportion of patients receiving neoadjuvant therapy (**Tables 1**, **2**). Eight patients in the C-BR group received radiotherapy. Eight patients in the SIE-BR group received radiotherapy. Fourteen patients in the SI-BR group received radiotherapy. There were no statistical differences between the three groups (**Table 1**).

**Table 2:** Perioperative features of patients.

Variable	PIBR		SIBR (N= 47)	P Variable
	C-BR (N= 51)	SIE-BR (N= 38)		
Operation time (min)				
Mean Range	110.00 (87.50,120.00)	142.50 (120.00,175.00)	120 (110,135)	0.000
Intraoperative blood loss (mL)				
Mean Range	50 (35,60)	50 (50,65)	65 (50,100)	0.000
Total drainage volume (mL)				
Mean Range	198.25±44.662	209.08±46.158	278.09±67.642	0.000
Hospitalization cost (yuan)				
Median Q1–Q3	44180.3000 (41584.4850,58762.0400)	43436.5600 (41917.1200,45778.2200)	43335.9400 (39868.1650,46846.1150)	0.081

SIBR subpectoral Implants for Breast Reconstruction; PIBR prepectoral Implants for Breast Reconstruction;SIE-BR single-port insufflation endoscopic for breast reconstruction; C-BR conventional breast reconstruction.

### 3.2 Comparison of perioperative characteristics

In the C-BR group, 45 patients underwent sentinel lymph node dissection (SLND), and 6 patients underwent SLND combined with axillary lymph node dissection (ALND). In the SIE-BR group, 30 patients underwent SLND, and 8 patients underwent SLND combined with ALND. In the SI-BR group, 33 patients underwent SLND, and 14 patients underwent SLND combined with ALND. There were no statistically significant differences among the three groups (**Table 1**). There were significant differences in surgical time among the three groups. The C-BR group had the shortest surgical time, with a median of 110.00 (87.50, 120.00) minutes, while the SIE-BR group had the longest surgical time, with a median of 142.50 (120.00, 175.00) minutes, p < 0.001 (**Table 2**). There were also significant differences in intraoperative blood loss among the three groups. The C-BR group had the least blood loss, with a median of 50 (35, 60) ml, while the SIE-BR group had the most blood loss, with a median of 65 (50, 100) ml, p < 0.001 (**Table 2**). The total postoperative drainage volume varied significantly among the three groups. The C-BR group had the least drainage volume, with a mean of 198.25 ± 44.662 ml, while the SIE-BR group had the most drainage volume, with a mean of 278.09 ± 67.642 ml, p < 0.001 (**Table 2**).

### 3.3 Postoperative complications and oncological outcomes

Patients were divided into the prepectoral implant group (PIBR) with 89 individuals and the subpectoral implant group (SIBR) with 47 individuals to observe postoperative complications. No patients experienced implant loss or flap necrosis. In the PIBR group, 1 patient developed a wound infection, while 4 patients in the SIBR group developed a wound infection. The incidence of wound infection was significantly lower in the PIBR group compared to the SIBR group (1.12% vs 8.51%, P < 0.05). The PIBR group had 4 patients with capsular contracture, all of which were mild. In the SIBR group, 7 patients experienced capsular contracture, including 5 with mild and 2 with severe contracture. The incidence of capsular contracture was significantly lower in the PIBR group compared to the SIBR group (4.49% vs 14.89%, P < 0.05). The PIBR group had 2 cases of postoperative wound dehiscence, while the SIBR group had 1 case. The incidence of wound dehiscence was statistically similar between the two groups. The PIBR group had 2 patients with severe chest muscle pain, while the SIBR group had 7 patients with severe chest pain. The incidence of chest muscle pain was significantly lower in the PIBR group compared to the SIBR group (2.25% vs 14.8%, P < 0.05). The incidence of implant rippling was statistically similar between the two groups (**Table 3**).

**Table 3:** Postoperative complications.

			P Variable
	PIBR (N= 89)	SIBR (N= 47)	
wound infection	1.12% (n=1)	8.51% (n=4)	0.048
Incision dehiscence	2.25% (n=2)	2.13% (n=1)	0.346
capsular contracture	4.49% ( n=4)	14.89% ( n=7)	0.048
chest myalgia	2.25% ( n=2)	14.8% (n=7)	0.014
severe radial fold	3.37% ( n=3)	6.38% ( n=3)	0.708
mile radial fold	7.87% ( n=7)	6.38% ( n=3)	1.000

SIBR subpectoral Implants for Breast Reconstruction; PIBR prepectoral Implants for Breast Reconstruction; SIE-BR single-port insufflation endoscopic for breast reconstruction; C-BR conventional breast reconstruction.

As of June 2024, only 1 patient in the PIBR group experienced axillary lymph node recurrence, so no further analysis was performed.

### 3.4 BREAST-Q questionnaire results

All patients were assessed using the BREAST-Q questionnaire at 6 months postoperative for subjective measurements of psychological well-being, physical health (chest function protection), and sexual health. Patients who underwent prepectoral implant reconstruction reported significantly higher satisfaction scores in chest area physical health and psychological well-being. Satisfaction with breast appearance and sexual health was statistically similar between the two groups (**Table 4**).

**Table 4:** BREAST−Q scale scores of patients.

			P Variable
	PIBR (N= 89)	SIBR (N= 47)	
Physical well-being: chest	29 (25,30)	22 (19,26)	0.000
Psychological well-being	44 (42,45)	41 (38,44)	0.000
Satisfaction with breasts	58.97±2.19	8.68±5.08	0.111
Sexual well-being	21 (20,22)	22 (20,23)	0.056

BREAST-Q® version 2.0 © Memorial Sloan Kettering Cancer Center and The University of British Columbia, 2017. SIBR, subpectoral Implants for Breast Reconstruction; PIBR, prepectoral Implants for Breast Reconstruction; SIE-BR, single-port insufflation endoscopic for breast reconstruction; C-BR, conventional breast reconstruction.

## 4 Discussion

With continuous advancements in breast cancer screening, diagnosis, and treatment, the overall prognosis for breast cancer patients has significantly improved, leading to higher patient expectations for postoperative aesthetic outcomes and quality of life. Under the premise of effective treatment, addressing how to repair functional and aesthetic deficiencies caused by surgery to reduce the physical and emotional trauma of breast loss has become an important consideration for clinicians in the treatment of breast cancer patients (16). Implant-based breast reconstruction has become the most popular choice for breast reconstruction after mastectomy due to its relatively low risk of secondary damage and simpler surgical difficulty. However, the debate over the choice of implant placement plane in reconstruction has never ceased. This study included 136 patients undergoing implant-based breast reconstruction after mastectomy. Both techniques using the prepectoral approach have the advantages of low complication rates, good aesthetic outcomes, and reliable tumor safety in mid-term follow-up, making them ideal options for breast reconstruction.

Since the first reported case in the 1970s, breast reconstruction approaches have evolved from prepectoral to subpectoral and back to prepectoral (17, 18). Each approach has its own advantages and disadvantages in terms of safety, accessibility, and aesthetic outcomes. In the 1970s, Snyderman and Guthrie (19) first reported a technique for placing implants directly under the skin after mastectomy. This technique was gradually abandoned due to issues such as thin skin flaps, insufficient subcutaneous tissue, and complications like infection, capsular contracture, wound dehiscence, and implant exposure.

To address these issues, Radovan (20) pioneered the subpectoral implant placement technique in the 1980s. This method, which provides sufficient soft tissue coverage over the implant, significantly reduced the rates of infection, flap necrosis, capsular contracture, and implant exposure. It also avoided implant displacement caused by subcutaneous placement. Despite its widespread use, the subpectoral approach has some drawbacks. It requires dissection of the pectoralis major and minor muscles, which may result in chest wall pain, movement restrictions, skin “wrinkling,” and muscle contraction leading to breast deformity. Additionally, radiation-induced fibrosis can cause implant displacement, and severe capsular contracture may even lead to implant removal (21–24). The limited space in the subpectoral area also restricts the implant volume and the reconstruction of breast shape, and the anatomical structure of the pectoralis major can result in inadequate coverage on the lateral and inferior aspects of the implant, leading to palpable implants (25). In this study, the surgeons tailored the implant coverage based on specific conditions. For patients with small to medium-sized breasts and smaller implants, adjacent autologous fascia (latissimus dorsi, serratus anterior, and pectoral fascia) was extended to cover the lower part of the implant. For larger or more drooping implants, the pectoralis major was detached from its rib attachment, and adjacent autologous fascia or synthetic patches were used to connect the detached pectoralis major to the inframammary fold, ensuring complete coverage of the implant and maintaining the continuity of the breast’s lateral contour. This approach resulted in better aesthetic outcomes for breast reconstruction. This study also confirmed that, after using the above method, patient satisfaction with breast appearance and sexual health in the PIBR and SIBR groups was statistically similar (**Table 4).** The key to retaining the autologous fascia is preserving the pectoral fascia, which contrasts with the traditional requirement to remove the pectoral fascia during radical mastectomy (26). Current research suggests that preservation of the pectoral fascia in modified radical mastectomy does not correlate with local recurrence or patient prognosis (27, 28), confirming the safety of using the pectoral fascia to cover implants. However, some studies have short follow-up periods and small sample sizes, so this method should be applied with caution in clinical practice. It is advisable to select patients with a lower risk of recurrence and ensure that the pectoral fascia is not invaded by cancerous tissue.

In recent years, advancements in understanding breast anatomy and the application of new technologies and materials have brought prepectoral implant-based reconstruction back into the spotlight (29–31). This approach has significantly improved upon the drawbacks of subpectoral implant-based reconstruction, such as chest wall pain, restricted movement, and capsular contracture (18). However, complications such as implant visibility and rippling still exist due to the lack of tissue coverage, and the risk of wound dehiscence and implant exposure remains relatively high with traditional open prepectoral approaches. With the widespread adoption of endoscopic techniques, endoscopic or robotic-assisted breast mastectomy and implant reconstruction have become emerging trends. These techniques offer similar tumor safety as traditional open surgery while providing advantages such as concealed incisions and no visible skin surface cuts, resulting in better cosmetic outcomes. They also protect flap blood supply, thereby reducing the occurrence of wound dehiscence and implant exposure (32–34). Traditionally, it was believed that the breast lacks natural cavities, and a single axillary incision limited the surgical field, increasing surgery time and difficulty. However, in this study, the surgeons utilized cold-knife dissection of the skin flaps and glandular tissue, opting for gas-inflated techniques to create the breast retropectoral space and dissection of the gland and surrounding ligaments. This approach not only protected the flaps from thermal damage but also reduced surgery time. After the learning curve, the perioperative characteristics and postoperative cosmetic results of the endoscopic method were found to be comparable to those of open surgery.

One of the key goals of implant-based breast reconstruction is to improve patients’ quality of life. Tools such as the BREAST-Q scale, which measures satisfaction with breast appearance, satisfaction with outcomes, psychosocial well-being, sexual well-being, and physical well-being, enable direct comparison of the impact of different reconstruction methods on patient satisfaction and quality of life. Research has indicated that satisfaction scores on most BREAST-Q modules are similar for both prepectoral and subpectoral implant-based breast reconstruction (35). This study found that the PIBR group had significantly higher satisfaction scores in terms of physical and psychological well-being related to the chest area. The satisfaction with breast appearance and sexual health was statistically similar between the two groups. This suggests that prepectoral implant-based breast reconstruction offers better psychological and physiological health, contributing to improved overall quality of life for patients.

### 4.1 Postoperative complications and prognosis analysis

The overall infection rate was 3.7%, with 1 case in the PIBR group and 4 cases in the SIBR group. The infection rate in the PIBR group was significantly lower than that in the SIBR group. However, all these patients, despite receiving treatment for infection and wound care, did not experience severe complications such as implant removal.

The occurrence of capsular contracture within one year after surgery: The PIBR group had 4 cases of mild capsular contracture, with an incidence rate of 4%. In the SIBR group, the overall incidence of capsular contracture was 13%, including 5 cases of mild and 2 cases of severe capsular contracture. The incidence of capsular contracture in the PIBR group was significantly lower than in the SIBR group. This difference may be related to factors such as the avoidance of muscle movement-induced friction and pressure on the implant in the prepectoral approach, improvements in surgical techniques, better understanding of anatomical layers, and reduced damage to surrounding tissues. Literature also suggests that individual patient factors, including their response to surgery and implants, may influence the occurrence of capsular contracture (18).

The incidence of chest wall pain was significantly higher in the SIBR group compared to the PIBR group (14.8% vs 2.25%, P < 0.05). The incidence of rippling was 12.7% in the SIBR group and 11% in the PIBR group, with statistically similar rates. This finding differs from some existing literature (21–24), which may be due to subjective patient self-assessment or shorter follow-up times.

### 4.2 Perioperative characteristics analysis

In the C-BR group, 45 patients underwent sentinel lymph node dissection (SLND), and 6 patients underwent SLND combined with axillary lymph node dissection (ALND). In the SIE-BR group, 30 patients underwent SLND, and 8 patients underwent SLND combined with ALND. In the SI-BR group, 33 patients underwent SLND, and 14 patients underwent SLND combined with ALND (**Table 1**). There were significant differences in surgical time among the three groups. The C-BR group had the shortest surgical time, with a median of 110.00 (87.50, 120.00) minutes, while the SIE-BR group had the longest surgical time, with a median of 142.50 (120.00, 175.00) minutes, p < 0.001 (**Table 2**). There were also significant differences in intraoperative blood loss among the three groups. The C-BR group had the least blood loss, with a median of 50 (35, 60) ml, while the SIE-BR group had the most blood loss, with a median of 65 (50, 100) ml, p < 0.001 (**Table 2**). The total postoperative drainage volume varied significantly among the groups. The C-BR group had the least drainage volume, with a mean of 198.25 ± 44.662 ml, while the SIE-BR group had the most drainage volume, with a mean of 278.09 ± 67.642 ml, p < 0.001 (**Table 2**).

Only one patient in the PIBR group experienced axillary lymph node recurrence, so no further analysis was performed.

### 4.3 Satisfaction analysis

Subjective measurements of breast satisfaction, psychosocial status, chest wall condition, and sexual health were assessed using the BREAST-Q questionnaire. Compared to patients undergoing subpectoral implant reconstruction, those receiving prepectoral implant reconstruction reported significantly higher satisfaction scores in chest area physical health and psychological well-being. Satisfaction with breast appearance and sexual health was statistically similar between the two groups (**Table 4**).

Under the premise of ensuring oncological safety, implant-based breast reconstruction achieved excellent aesthetic outcomes and improved patient quality of life. The prepectoral approach avoids complications such as chest wall pain, movement deformities, and shoulder mobility issues that are associated with the subpectoral approach, and it has a lower rate of capsular contracture (5, 36–39). The prepectoral approach better aligns with the natural breast structure, provides improved droop, enhances patient comfort, and offers better postoperative aesthetic outcomes, thereby contributing to an improved quality of life for patients.

## 5 Conclusion

In summary, prepectoral implant-based breast reconstruction has advantages over open subpectoral reconstruction in terms of aesthetics and preservation of patients’ physical function, without increasing the patient’s financial burden. The C-BR procedure has a shorter operative time, providing a certain cost advantage. SIE-BR offers a scar-free breast surface, lower risk of incision dehiscence, and good aesthetic outcomes. Prepectoral implant-based breast reconstruction truly achieves a balance of aesthetics, safety, and operability, and has broad potential for application among patients who meet the surgical indications. It is worth promoting. However, this study has certain limitations. As a retrospective study with a relatively short follow-up period, its conclusions need to be validated by large-scale, long-term prospective research.

## Data Availability

The raw data supporting the conclusions of this article will be made available by the authors, without undue reservation.
